# Renal Blood Flow as Marker for Disease Progression in ADPKD

**DOI:** 10.1016/j.ekir.2026.107050

**Published:** 2026-09-01

**Authors:** Rijk Wagenaar, Abdulfataah A.A. Mohamed, Esther Meijer, Martin H. De Borst, Maatje A. van Gastel, Lianne A. Messchendorp, Ron T. Gansevoort, Thomas Bais, DIPAK consortium, J.P.H. Drenth, R.T. Gansevoort, M.D.A. van Gastel, E. Meijer, L. Messchendorp, T. Nijenhuis, T. Bais, D.J.M. Peters, M. Salih, S. Spijker

**Affiliations:** 1 Division of Nephrology, Department of Internal Medicine, University Medical Cencer Groningen, University of Groningen, Groningen, The Netherlands; 2 Department of Clinical Pharmacy and Pharmacology, University Medical Center Groningen, University of Groningen, Groningen, The Netherlands

**Keywords:** autosomal dominant polycystic kidney disease, disease progression, renal blood flow

## Abstract

**Background:**

Disease progression in autosomal dominant polycystic kidney disease (ADPKD) is variable. Renal blood flow (RBF) and filtration fraction (FF), reflecting hemodynamic alterations and hyperfiltration, may improve risk stratification.

**Methods:**

This prospective study included 238 adults with ADPKD from the Developing Intervention Strategies to Halt Progression of ADPKD observational cohort study and 1421 healthy controls. Multivariable regression analyses assessed associations of RBF and FF with disease severity and progression. Kaplan-Meier analysis and classification performance metrics were used to evaluate clinical use beyond established risk stratification tools.

**Results:**

Cross-sectionally, patients with ADPKD had significantly lower RBF compared with controls across all age groups, even in young patients with similar estimated glomerular filtration rate (eGFR) to controls. Within patients with ADPKD, lower RBF was associated with reduced kidney function and larger kidney volume. During follow-up of 5.6 ± 3.5 years, higher RBF (per SD) was associated with slower annual eGFR decline (standardized β = 0.34; 95% confidence interval [CI], 0.09–0.58; *P* = 0.01), lower odds of rapid disease progression (eGFR decline ≥ 3.0 ml/min per 1.73 m^2^/year) (odds ratio [OR] = 0.34; 95% CI, 0.17–0.70; *P* = 0.003) and lower composite-end point risk (kidney failure or ≥ 30% eGFR decline) (hazard ratio [HR] = 0.35; 95% CI, 0.20–0.60; *P* < 0.001). FF was significantly higher in patients aged 30–40 years, consistent with early hyperfiltration, but was not associated with disease progression. For the association with rapid progression, adding RBF to the Mayo Imaging Classification (MIC) increased the area under the curve (AUC) from 0.69 (95% CI, 0.60–0.76) to 0.79 (95% CI, 0.72–0.85).

**Conclusion:**

Reduced RBF is an early hallmark of ADPKD, associated with disease severity and kidney function decline, and improves risk stratification beyond MIC.

## Graphical abstract

**Figure:**
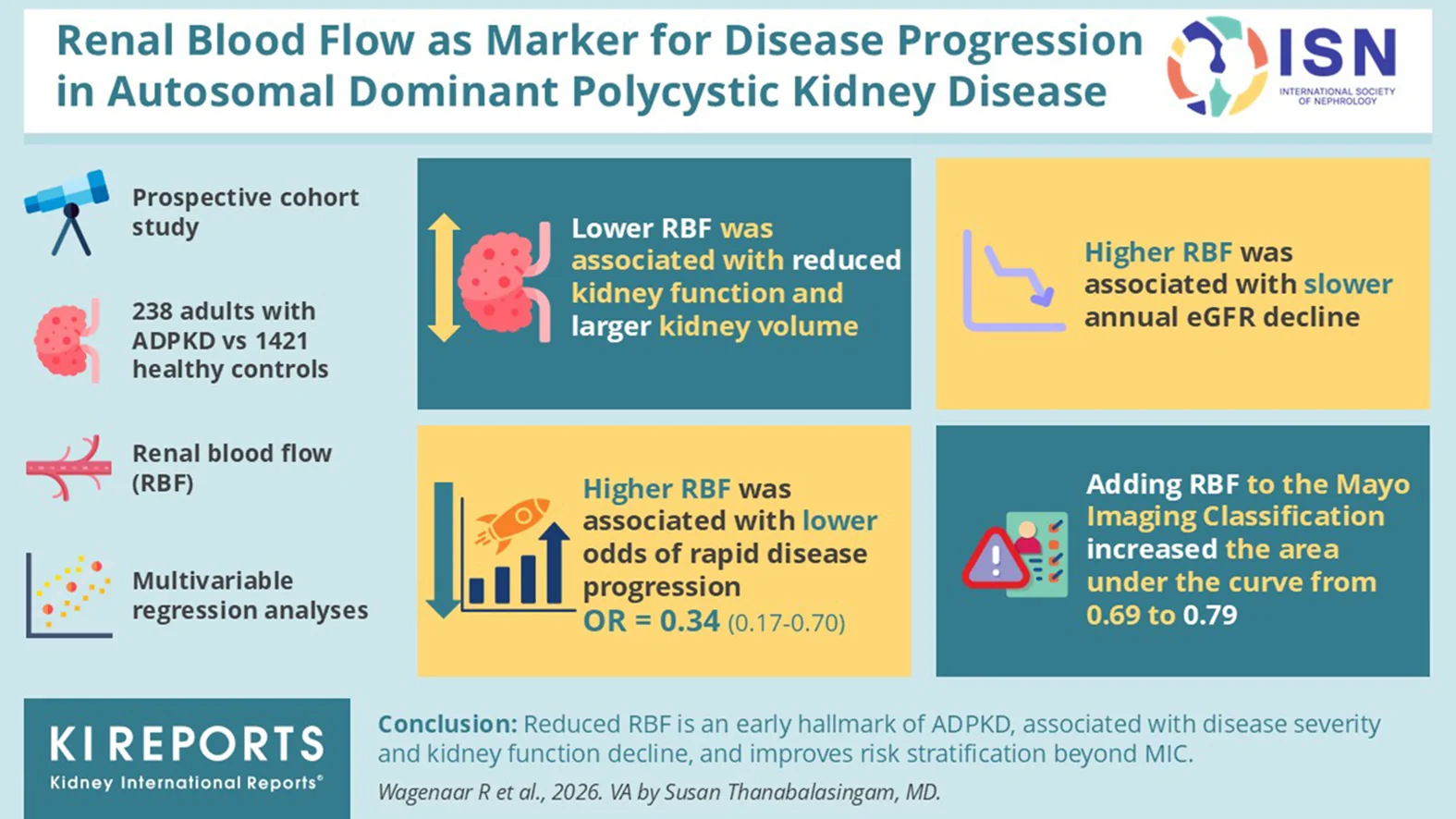

ADPKD is the most common hereditary kidney disorder, with a prevalence of approximately 1 in 1000 individuals.^1^ ADPKD is characterized by the continuous formation and gradual growth of kidney cysts, leading to an increase in kidney volume of up to 7 litres per kidney, and progressive impairment of kidney function.^2^ Around 70% of affected patients will eventually develop kidney failure.^3^ The rate of progression varies widely, even within families.^4^^,^^5^ Tolvaptan can significantly slow disease progression in patients at high risk for rapid disease progression, but side effects and costs limit its use and make accurate risk stratification essential.^6^^,^^7^ Risk for rapid disease progression is currently estimated mainly using the MIC on the basis of total kidney volume adjusted for height and age.^8^ Although this tool is valuable at a population level, its accuracy for individual patients is limited. The MIC is associated with heterogeneity in rate of eGFR decline within subclasses, particularly in patients with MIC 1C, leading European guidelines to require additional evidence to allow defining a high risk of rapid disease progression that qualifies for initiating tolvaptan.^7^^,^^9^ This limitation highlights the need for additional markers to improve risk stratification.^7^

RBF and FF, reflecting kidney hemodynamic function and hyperfiltration, have been proposed as potential markers of disease severity and progression in ADPKD.^10^, ^11^, ^12^, ^13^ Progressive cyst growth compresses intrarenal blood vessels, reducing kidney perfusion, whereas vascular dysfunction further decreases RBF.^14^ This triggers compensatory hyperfiltration and increased intraglomerular pressure.^15^ Previous studies have shown that RBF is reduced and FF increased in patients with early stage ADPKD, and that these measures are associated with kidney size and kidney function.^11^, ^12^, ^13^ Longitudinal data from 131 participants with measurements of RBF in the Consortium for Radiologic Imaging Studies of Polycystic Kidney Disease showed that RBF reduction paralleled volume increase, preceded GFR decline, and predicted disease progression after adjustment for multiple clinical covariates.^11^ However, these analyses could not be adjusted for MIC because it had not yet been developed. More recently, a larger longitudinal study confirmed that lower RBF is associated with kidney function decline and progression to kidney failure after adjustment for several clinical covariates, but the value beyond established risk stratification tools, such as the MIC, was not investigated in these analyses.^10^ Consequently, the role of RBF as a prognostic marker beyond current risk models remains unclear. This uncertainty is explicitly recognized in the Kidney Disease: Improving Global Outcomes 2025 Clinical Practice Guideline for ADPKD, which calls for further research into the prognostic value of RBF.^16^

This study aims therefore to determine whether RBF is already reduced and hyperfiltration (expressed as FF) is present in patients with early-stage ADPKD. In addition, it will investigate whether RBF and FF are associated cross-sectionally with disease severity and longitudinally with kidney function decline. Finally, the additional prognostic value of these measures beyond existing prognostic tools will be investigated.

## Methods

### Study Population

This study used data from the ongoing Developing Intervention Strategies to Halt Progression of ADPKDobservational study, a multicenter prospective cohort study that studies natural disease progression in patients with ADPKD in the Netherlands. We included 238 patients enrolled at the University Medical Center Groningen that underwent systemic effective renal plasma flow (ERPF) and FF measurements. All patients were aged ≥18 years and diagnosed with ADPKD according to the Pei-Ravine criteria or by genetic testing.^17^ Patients had yearly eGFR assessments and triennial measurements of height-adjusted total kidney volume (HtTKV), measured GFR (mGFR), and ERPF. Patients using tolvaptan or somatostatin analogues at baseline were excluded. Patients who initiated tolvaptan during follow-up were included in the analyses with their data collected before treatment initiation, with follow-up censored at the time of treatment start to minimize potential selection bias. Patients with an eGFR ≤ 15 ml/min per 1.73 m^2^ or kidney replacement therapy (kidney transplantation or dialysis) were excluded from study participation, as well as patients with other kidney diseases or systemic diseases that affect kidney function. A schematic overview of patient selection is presented in Supplementary Figure S1. Approval for the Developing Intervention Strategies to Halt Progression of ADPKD observational study was obtained from the institutional review board of the University Medical Center Groningen (METC, 2013/040), and the study complied with International Conference on Harmonization Good Clinical Practice guidelines.

Living kidney donors before donation served as the healthy control group. Data were obtained from the TransplantLines Biobank and Cohort Study. This ongoing prospective study invites all patients and donors (≥ 18 years) undergoing solid organ transplantation at the University Medical Center Groningen since 2015. Donors with kidney disease or systemic diseases that affect kidney function were excluded. The study was registered at ClinicalTrials.gov (NCT03272841) and approved by the local institutional review board (METC 2014/077). All participants in both groups gave written informed consent. Both studies are conducted in compliance with the WMA Declaration of Helsinki and the Declaration of Istanbul.^18^^,^^19^

### Measurements and Calculations

In patients with ADPKD, blood pressure was measured at rest, 3 times at 2 minute intervals using an automatic device. Hemoglobin and creatinine were measured from blood and serum samples using standard laboratory techniques. Total kidney volume (TKV) was measured using an automated stereological method, by a team in the University Medical Center Groningen specifically trained for this purpose, as described previously.^20^ TKV was divided by height to calculate HtTKV, which was indexed for age to assign the MIC class.^8^

For both, patients with ADPKD and living kidney donors, kidney function was assessed by measuring mGFR and ERPF using constant infusion of ^125^I-iothalamate and ^131^I-Hippuran (Supplementary Methods), as previously described.^21^ eGFR was calculated using the Chronic Kidney Disease–Epidemiology Collaboration equation 2009, which is the standard equation used in routine clinical practice across Europe.^22^^,^^23^ mGFR was normalized to body surface area, estimated with the Mosteller formula.^24^ Hematocrit was derived from hemoglobin using the formula Hct = 0.0485·Hb(mmol/L)+0.0083.^25^ RBF and FF were calculated as RBF (mL/min) = $\frac{ERPF\;\left(\text{mL}/\min\right)}{\left(1-Hct\right)}$ and FF = $\frac{mGFR\;\left(\text{mL}/\min\right)}{ERPF\;\left(\text{mL}/\min\right)}$, respectively.^26^

### Statistical Analyses

#### Comparison Between Patients With ADPKD and Healthy Controls

Statistical analyses were performed using SPSS version 28.0 (IBM Corp, Armonk, NY, USA). Kaplan-Meier curves and discriminative analyses, including Receiver Operating Characteristic curves, Concordance index, and Net Reclassification Improvement, were performed in R (version 4.3.2) using RStudio (version 2024.04.2+764). Baseline clinical characteristics of patients with ADPKD and healthy controls were summarised using descriptive statistics. Normally distributed variables were expressed as mean ± SD, nonnormally distributed variables were expressed as median (IQR). A *P-*value < 0.05 was considered statistically significant.

To characterise age-related differences in kidney hemodynamics, patients with ADPKD and healthy controls were stratified into predefined age categories (<30, 30–40, 40–50, 50–60, and >60 years). Within each age group, mean values of RBF, eGFR, mGFR, and FF were compared between patients and controls using independent *t*-tests. These analyses were conducted to evaluate whether differences in kidney hemodynamic parameters were already present in early adulthood, and whether the magnitude of these differences varied across age categories.

#### Associations With Disease Progression

Among patients with ADPKD, univariable and multivariable linear regression analyses were used to assess associations of RBF and FF with baseline eGFR, mGFR, and HtTKV, as well as with eGFR slope. Continuous variables were *z*-score standardized by subtracting the mean and dividing by the SD. The standardized β represents the change in the outcome associated with a 1 SD increase in the predictor, facilitating comparison of effect sizes across variables with varying units and scales. Because eGFR slopes are negative, a higher slope (e.g., −2 vs. −4 ml/min per 1.73 m^2^year) indicates slower kidney function decline. Annual eGFR slope was estimated from longitudinal eGFR measurements using linear mixed-effects models, including random effects for patients and time and an unstructured covariance structure. Logistic regression was applied to investigate univariable and multivariable associations with rapid disease progression. Rapid disease progression was defined as an eGFR decline ≥ 3.0 ml/min per 1.73 m^2^/year, because this threshold is used in treatment guidelines to identify patients eligible for initiation of tolvaptan.^7^ Cox proportional hazards regression was used to assess univariable and multivariable associations with the combined end point of kidney failure or a ≥30% decline in eGFR from baseline. Kidney failure was defined as initiation of dialysis, kidney transplantation or an eGFR ≤ 15 ml/min per 1.73 m^2^. Multivariable models were adjusted for established clinical risk factors, including sex, age, baseline eGFR or baseline mGFR, and MIC.^8^^,^^27^

To assess discriminative performance for rapid disease progression, Receiver Operating Characteristic curves were generated for the MIC, RBF, and models that additionally incorporated RBF. AUC with 95% CI were obtained using nonparametric bootstrapping with 1000 iterations. Likelihood ratio tests were used to compare logistic regression models. To evaluate improvement in risk classification beyond the main model, the Net Reclassification Improvement was calculated, with a binary probability cut-off of 0.5 (≥0.5 and <0.5 for predicted rapidly- and slowly progressive disease, respectively).^28^

For the time to event outcome, Kaplan-Meier curves were generated on the basis of the tertiles of RBF and FF. Differences between groups were assessed using log-rank tests. Discrimination of the Cox models was quantified using the Concordance index with 95% CI derived from 1000 bootstrap iterations.^29^

Several subgroup were performed, i.e., patients aged <40 years and in those with MIC 1C, as risk stratification is particularly challenging in those groups. In these subgroups, the regression analyses were repeated using the same strategy as in the primary ADPKD cohort. Furthermore, analyses were repeated using mGFR instead of eGFR to evaluate whether associations were consistent when using measured kidney function. Sensitivity analyses included assessing RBF corrected for body surface area. In addition, a sensitivity analysis including pathogenic variant type alongside MIC in the models was performed.

## Results

### Baseline Characteristics

A total of 238 patients with ADPKD and 1421 healthy kidney donors were included. Patients with ADPKD had a mean age of 44.4 ± 12.4 years and a baseline eGFR of 70.0 ± 30.9 ml/min per 1.73 m^2^. In this group, 59.7% were female. Mean RBF and FF were 453 ± 190 ml/min and 0.32 ± 0.04, respectively. Healthy kidney donors had a mean age of 55.3 ± 11.6 years and 54.0% were female. Mean baseline eGFR in this group was 87.9 ± 14.4 ml/min per 1.73 m^2^, and mean RBF and FF were 627 ± 149 ml/min and 0.32 ± 0.04, respectively (Table 1).

**Table 1:** Baseline characteristics of patients with ADPKD and healthy controls

Variable	Patients with ADPKD (*n* = 238)	Healthy controls (*n* = 1421)
Female sex, No. (%)	142 (59.7)	768 (54.0)
Age, yr	44.4 ± 12.4	55.3 ± 11.6
Ethnicity, No. (%)		
Caucasian	224 (94.1)	1073 (75.5)
Asian	4 (1.7)	113 (8.0)
Other	3 (1.3)	11 (0.8)
Height, m	1.77 ± 0.1	1.74 ± 0.1
Weight, kg	83.5 ± 17.8	80.0 ± 13.3
BMI, kg/m^2^	26.7 ± 5.1	26.2 ± 3.4
BSA, m^2^	2.0 ± 0.2	2.0 ± 0.2
Systolic BP, mm Hg	128.4 ± 11.5	126.9 ± 13.7
Diastolic BP, mm Hg	79.0 ± 9.2	75.3 ± 9.4
RAAS inhibitor use, No. (%)	135 (56.7)	104 (7.3)
Hemoglobin, mmol/l	8.5 ± 0.9	8.9 ± 0.8
Hematocrit, L/L	0.42 ± 0.04	0.44 ± 0.04
Creatinine, μmol/l	116.0 ± 64.6	75.6 ± 13.8
eGFR, ml/min per 1.73 m^2^	70.0 ± 30.9	87.9 ± 14.4
mGFR, ml/min per 1.73 m^2^	72.2 ± 29.3	95.1 ± 16.4
RBF, ml/min	453 ± 190	627 ± 149
FF	0.32 ± 0.04	0.32 ± 0.04
HtTKV, ml/m	1046 ± 915	-
*PKD*-variant, No. (%)		
PKD2	40 (16.8)	-
PKD1 nontruncating	80 (33.6)	-
PKD1 truncating	100 (42.0)	-
MIC, No. (%)		
1A	8 (3.4)	-
1B	39 (16.4)	-
1C	77 (32.4)	-
1D	45 (18.9)	-
1E	21 (8.8)	-
2 (atypical)	7 (2.9)	-
Missing	41 (17.2)	-

MI, body mass index; BP, blood pressure; BSA, body surface area; eGFR, estimated glomerular filtration rate; FF, filtration fraction; HtTKV, height-adjusted total kidney volume; MIC, Mayo Imaging Classification; mGFR, measured glomerular filtration rate; PKD, polycystic kidney disease; RAAS, renin-angiotensin-aldosterone system; RBF, renal blood flow.

Data are presented as mean ± SD or number (%) unless otherwise indicated. To convert serum creatinine from μmol/l to mg/dl, divide by 88.42.

### Cross-Sectional Analyses

#### Hemodynamic Parameters in Patients With ADPKD and Healthy Controls

In individuals aged <30 years, RBF was already lower in patients with ADPKD compared with controls (−14%, *p* = 0.004), whereas eGFR did not differ significantly between groups (−3%, *P* = 0.3) and mGFR was only slightly lower in this age group (−6%, *P* = 0.04) compared with controls. From age 30 years onwards, eGFR, RBF, and mGFR in patients with ADPKD show increasing deviation at older age compared with healthy controls, because in both groups, RBF, eGFR, and mGFR declined with increasing age, but with more pronounced reductions in patients with ADPKD compared with controls. FF tended to be higher in patients with ADPKD aged <30 years and was significantly higher in those aged 30–40 years. In older age categories, FF did not differ significantly between patients and healthy controls (Figure 1).

**Figure 1:**
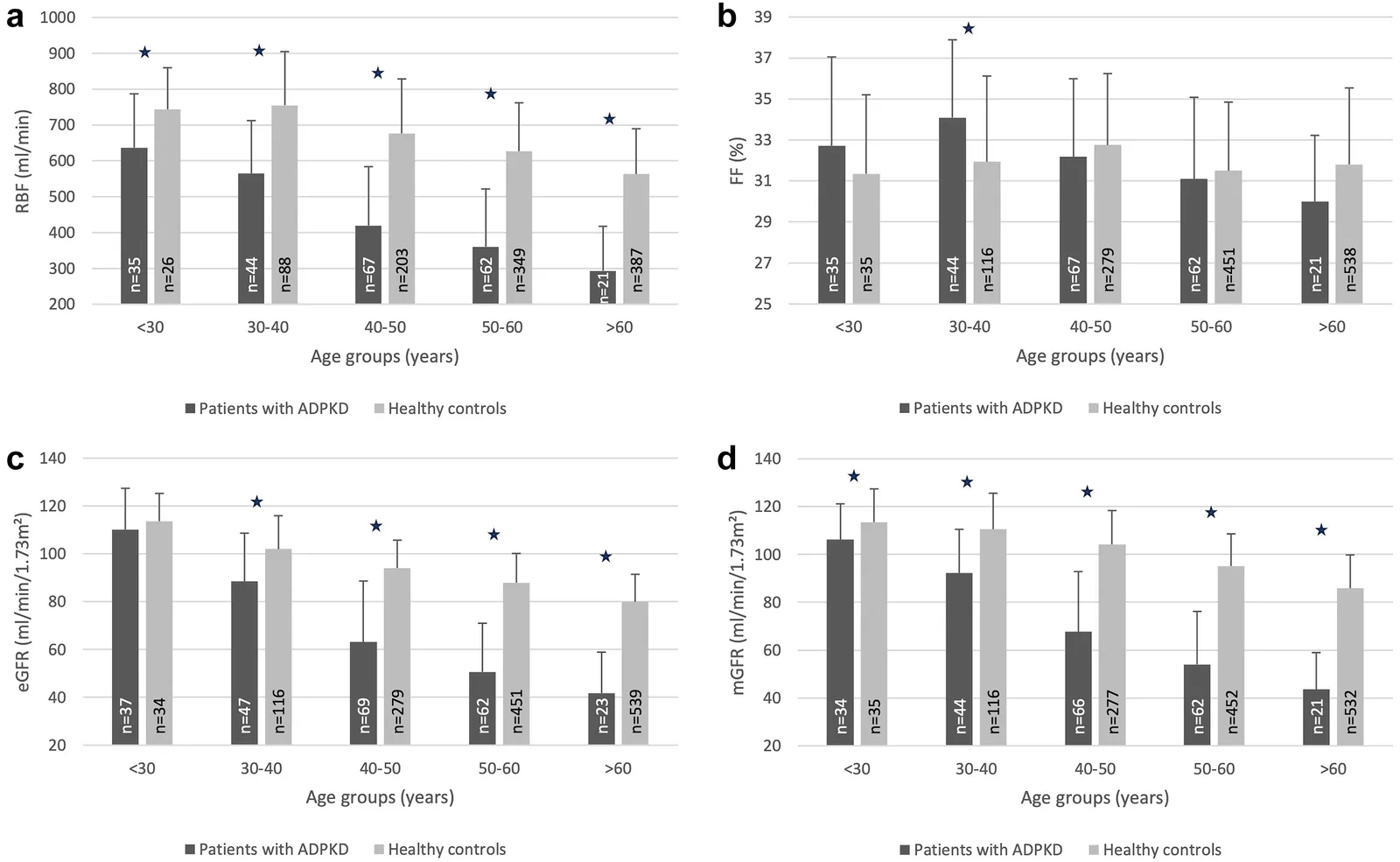
Kidney hemodynamic parameters in patients with ADPKD and healthy controls within age-subgroups. Mean values with SD of RBF, FF, eGFR and mGFR across age categories (<30, 30–40, 40–50, 50–60 and >60 years) in patients with ADPKD and healthy controls. For each parameter, patient and control values are shown within the same age group. Significant differences between patients and controls within an age group are indicated by ★ (*P* < 0.05). Sample sizes (*n*) are indicated within the bars. ADPKD, autosomal dominant polycystic kidney disease; RBF, renal blood flow; FF, filtration fraction; eGFR, estimated glomerular filtration rate; mGFR, measured glomerular filtration rate.

### Associations With Baseline eGFR, mGFR, and Kidney Volume in the ADPKD Cohort

In the univariate cross-sectional analyses, higher RBF was associated with better kidney function and smaller kidney volume. After adjustment for clinical covariates, RBF remained positively associated with baseline eGFR (St. β = 0.69; 95% CI, 0.60–0.74) and mGFR (St. β = 0.75; 95% CI, 0.66–0.81), and negatively associated with HtTKV (St. β =-0.19; 95% C,: −0.29 to −0.06) (all *P* < 0.05). FF was positively associated with baseline eGFR (St. β = 0.10; 95% CI, 0.02–0.19; *P* = 0.02) and mGFR (St. β = 0.20; 95% CI, 0.12–0.29; *P* < 0.001) in the adjusted model, whereas no significant association with HtTKV was observed in either model (Table 2).

**Table 2:** Cross-sectional associations of RBF and FF with baseline eGFR, mGFR and height-adjusted total kidney volume (HtTKV) in patients with ADPKD (*n* = 238)

Variable	Model	eGFR		mGFR		HtTKV	
		St. β-estimate (95% CI)	*P-*value	St. β-estimate (95% CI)	*P-*value	St. β-estimate (95% CI)	*P-*value
RBF	1	0.85 (0.76–0.90)	<0.001	0.86 (0.78–0.91)	<0.001	−0.36 (−0.44 to −0.20)	<0.001
	2	0.69 (0.60–0.74)	<0.001	0.75 (0.66–0.81)	<0.001	−0.19 (−0.29 to −0.06)	0.003
FF	1	0.27 (0.15–0.40)	<0.001	0.36 (0.24–0.48)	<0.001	−0.13 (−0.24 to 0.01)	0.07
	2	0.10 (0.02–0.19)	0.02	0.20 (0.12–0.29)	<0.001	−0.09 (−0.16 to 0.01)	0.07

CI, confidence interval; eGFR, estimated glomerular filtration rate; FF, filtration fraction; HtTKV, height-adjusted total kidney volume; mGFR, measured glomerular filtration rate; RBF, renal blood flow.

Model 1, univariate. Model 2, adjusted for sex, baseline age and Mayo imaging classification. All continuous variables were *z*-standardised. Standardized β represents the SD change in the outcome per 1-SD higher RBF or FF.

### Longitudinal Analyses

#### Disease Progression

Disease outcomes for the ADPKD cohort are summarized in Supplementary Table S1. The mean follow-up duration was 5.6 ± 3.5 years, and the mean annual eGFR slope was −2.79 ± 1.27 ml/min per 1.73 m^2^/year. Of 238 patients, 51 patients (21.4%)developed kidney failure (initiation of dialysis, kidney transplantation, or an eGFR ≤ 15 ml/min per 1.73 m^2^), and 111 of 238 (46.6%) reached the combined end point of 30% eGFR decline or kidney failure. Out of 238 patients, 112 (47.1%) and 126 (52.9%) of patients, respectively, were classified as rapid and slow progressions (annual eGFR loss of ≥3 or <3 ml/min per 1.73 m^2^). Rapid progressors showed, by definition, a faster rate of GFR loss.

#### Associations with subsequent eGFR slope

In the longitudinal analyses, higher baseline RBF was associated with a slower decline in eGFR slope in the primary cohort. In the unadjusted analyses (Table 3, Model 1), RBF showed a positive association with eGFR slope (St. β = 0.35; 95% CI, 0.23–0.47, *P* < 0.001), indicating that higher RBF was linked to slower kidney function decline. This association remained significant after adjustment for clinical covariates in Model 2 (St. β = 0.34; 95% CI, 0.09–0.58; *P* = 0.01). After substitution of baseline mGFR for eGFR in the multivariate model (Model 3), this association was no longer significant. FF was not significantly associated with eGFR slope in any of the models.

**Table 3:** Longitudinal associations of RBF and FF with kidney function outcomes in patients with ADPKD (*n* = 238)

Variable	eGFR slope		Rapidly progressive disease		Kidney failure or 30% eGFR decline	
	St. β-estimate (95% CI)	*P-*value	OR (95% CI)	*P-*value	HR (95% CI)	*P-*value
Primary cohort (*n* = 238)						
Model 1						
RBF	0.35 (0.23 to 0.47)	<0.001	0.41 (0.30–0.56)	<0.001	0.28 (0.21–0.37)	<0.001
FF	0.04 (−0.10 to 0.17)	0.6	0.95 (0.73–1.23)	0.7	0.80 (0.66–0.97)	0.02
Model 2						
RBF	0.34 (0.09–0.58)	0.01	0.34 (0.17–0.70)	0.003	0.35 (0.20–0.60)	<0.001
FF	−0.03 (−0.15 to 0.09)	0.6	1.15 (0.82–1.60)	0.4	1.02 (0.81–1.27)	0.9
Model 3						
RBF	0.13 (−0.13 to 0.39)	0.3	0.44 (0.21–0.92)	0.03	0.48 (0.27–0.85)	0.01
FF	−0.09 (−0.21 to 0.04)	0.2	1.43 (0.99–2.06)	0.06	1.23 (0.98–1.56)	0.08

CI, confidence interval; eGFR, estimated glomerular filtration rate; FF, filtration fraction; HR, hazard ratio; mGFR, measured glomerular filtration rate; OR, odds ratio; RBF, renal blood flow.

Model 1, univariate. Model 2, adjusted for sex, baseline age, eGFR at baseline and Mayo Imaging Classification. Model 3, identical to Model 2 with substitution of baseline mGFR for baseline eGFR. All continuous variables were z-standardised. Standardized β represents the SD change in the outcome per 1-SD higher RBF or FF; a positive β for eGFR slope indicates slower decline. ORs and HRs are reported per 1-SD higher RBF or FF; values < 1 indicate lower odds or risk.

#### Associations With Subsequent Rapid Disease Progression

Within the full cohort, 112 of 238 patients (47.1%) were classified as having rapid disease progression. In unadjusted analyses (Model 1), lower RBF was associated with rapid disease progression (OR, 0.41; 95% CI, 0.30–0.56; *P* < 0.001). This association remained statistically significant after adjustment for clinical covariates in Model 2 and Model 3. FF was not significantly associated with rapid disease progression in any of the models (Table 3).

In the Receiver Operating Characteristic curve analyses (Figure 2), the MIC model yielded an AUC of 0.69 (95% CI, 0.60–0.76) for identifying rapid disease progression, with comparable numbers for RBF with an AUC of 0.73 (95% CI, 0.65–0.79). The addition of RBF to MIC improved the AUC to 0.79 (95% CI, 0.72–0.85). Addition of clinical variables (sex, baseline age, and eGFR) increased the AUC to 0.82 (95% CI, 0.75– 0.87). The addition of mGFR instead of eGFR along with the other clinical variables yielded a similar AUC of 0.83 (95% CI, 0.77–0.88).

**Figure 2:**
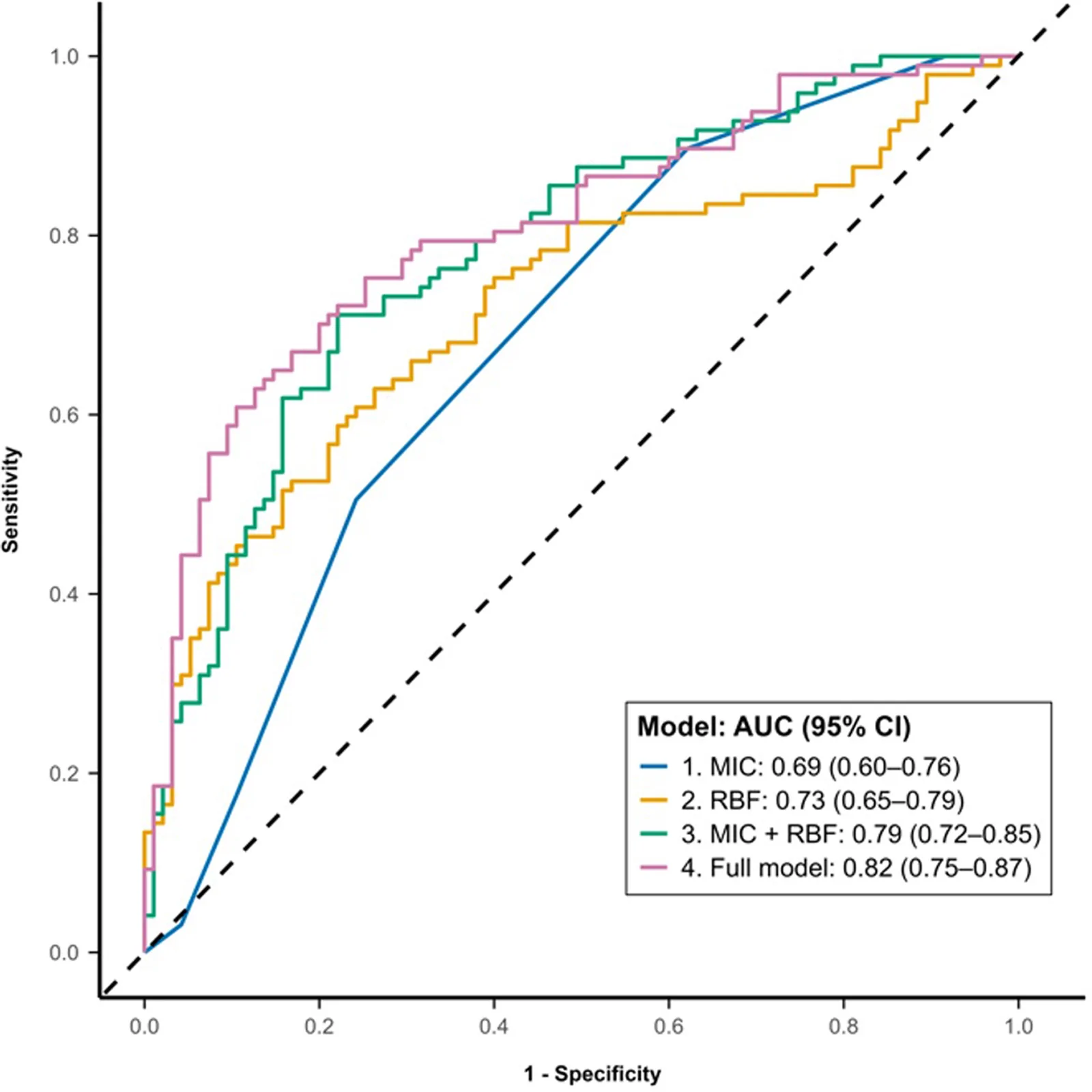
ROC curves for predicting rapid disease progression in the full ADPKD cohort (eGFR loss ≥ 3 ml/min per 1.73 m^2^/y). Receiver operating characteristic (ROC) curves are shown for models predicting rapid disease progression using (1) MIC alone; (2) RBF alone; (3) MIC plus RBF; and (4) the full model, including sex, age, baseline eGFR, MIC, and RBF. Corresponding AUC values with 95% CI are shown in the panel. ADPKD, autosomal dominant polycystic kidney disease; AUC, area under the curve; CI, confidence interval; MIC, Mayo Imaging Classification; RBF, renal blood flow; ROC, receiver operating characteristic.

Model fit increased according to the likelihood ratio test when RBF was added to the MIC model (LRT *P* < 0.001), the Net Reclassification Improvement of 17.5% (95% CI, 3.7–32.9) indicated that nearly 1 in 5 patients was more appropriately reclassified after inclusion of RBF.

#### Associations With Subsequent Incident Kidney Failure or ≥ 30% eGFR Decline

In the full cohort, 111 of 238 patients (46.6%) reached the combined end point of kidney failure or a ≥ 30% decline in eGFR. Kaplan-Meier analyses on the basis of tertiles of RBF showed significant differences, with lower RBF associated with shorter survival (log-rank *P* < 0.001) (Figure 3a). In contrast, no significant differences in survival were observed across tertiles of FF (log-rank *P* = 0.3) (Figure 3b).

**Figure 3:**
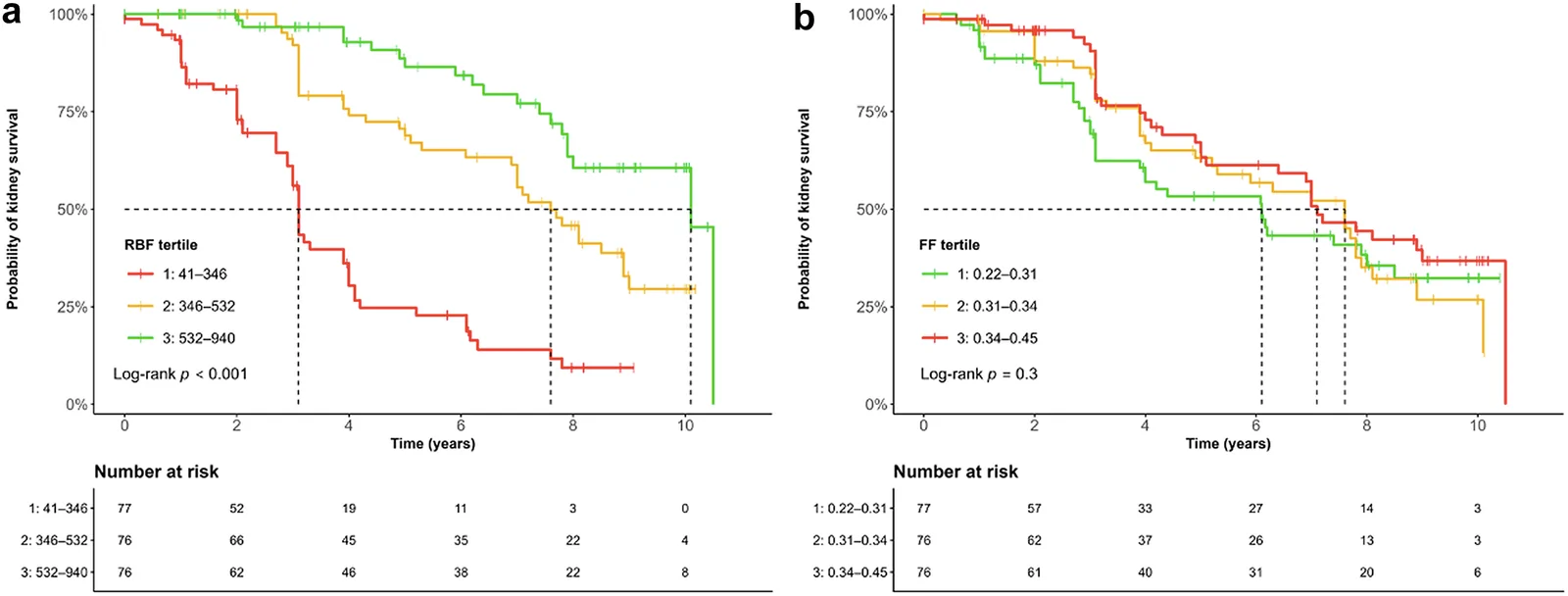
Kaplan-Meier curves for kidney survival according to tertiles RBF and FF. Kaplan-Meier curves showing kidney survival, defined as kidney failure or a ≥ 30% decline in eGFR, across tertiles of (a) RBF and (b) FF. Time on the x-axis reflects follow-up duration within the study. Tertile 1 corresponds to the lowest values and tertile 3 to the highest. Log-rank *P-*values are shown within each panel, and numbers at risk for each tertile are presented below the curves. ADPKD, autosomal dominant polycystic kidney disease; eGFR, estimated glomerular filtration rate; FF, filtration fraction; RBF, renal blood flow.

In unadjusted Cox regression analyses (Table 3, Model 1), higher baseline RBF was associated with a lower risk of the combined end point (HR, 0.28; 95% CI, 0.21–0.37, *P* < 0.001). This association remained statistically significant after adjustment for clinical covariates in Model 2 and after substitution of baseline mGFR for eGFR in Model 3. FF was also associated with a lower risk of the combined end point in unadjusted analyses (HR, 0.80; 95% CI, 0.66–0.97; *P* = 0.02), but this association did not remain significant after adjustment in Model 2 or Model 3. The C-index of the MIC model was 0.61 (95% CI, 0.56–0.67) and increased to 0.81 (95% CI, 0.77–0.85) after addition of RBF (LRT *P* < 0.001).

### Subgroup Analyses

The results for patients younger than 40 years (*n* = 84) and patients with MIC 1C (*n* = 77) are shown in Supplementary Tables S2 and S3. In patients younger than 40 years, baseline RBF was not significantly associated with eGFR slope in any of the models. In patients with MIC 1C, baseline RBF was positively associated with eGFR slope in unadjusted analyses. Although this association was attenuated and no longer statistically significant after adjustment for clinical covariates, the direction and magnitude of the effect estimates remained broadly similar with those observed in the overall cohort.

Among patients aged <40 years, 30 (35.7%) met criteria for rapid disease progression (eGFR decline ≥ 3.0 ml/min per 1.73 m^2^/year) and 22 (26.2%) reached the combined end point of kidney failure or a ≥ 30% decline in eGFR. In patients with MIC 1C, 38 (49.4%) met criteria for rapid disease progression and 43 (55.8%) reached the combined end point.

In unadjusted analyses, higher baseline RBF was associated with lower odds of rapid disease progression and a lower risk of the combined end point in both subgroups, with effect estimates comparable to those observed in the overall cohort. Similarly, after adjustment for the same set of clinical covariates, these associations did not remain statistically significant. FF was not significantly associated with eGFR slope, rapid disease progression, or the combined end point in any of the models in either subgroup.

### Sensitivity Analysis

Additional adjustment for pathogenic variant type in both the cross-sectional and longitudinal analyses did not materially change the observed associations (Supplementary Tables S4 and S5). Correction of RBF for body surface area likewise did not materially affect the results.

## Discussion

This study investigated RBF and FF in patients with ADPKD and their additional prognostic value beyond established risk stratification tools. We found that RBF was consistently associated with disease severity and progression. Compared with healthy controls, RBF was already reduced in young adults with ADPKD, whereas eGFR was similar. Lower RBF was associated with worse kidney function and larger kidney volume cross-sectionally, and with subsequent kidney function decline across different longitudinal kidney function outcomes. These associations were independent of established clinical risk factors including baseline kidney function. The incremental prognostic value improved substantially after adding RBF to the MIC, as reflected by improvements in AUC, Net Reclassification Improvement, and Concordance-index. An increased FF in patients aged 30–40 years indicated early hyperfiltration. The degree of hyperfiltration, however, was not associated with kidney volume or subsequent kidney function decline.

The cross-sectional findings are consistent with previous studies describing early hemodynamic alterations in ADPKD. Previous studies have reported evidence of hyperfiltration, as well as other early hemodynamic abnormalities in young or mildly affected individuals.^15^^,^^30^^,^^31^ However, the presence of hyperfiltration in early ADPKD remains debated. A study assessing kidney function reserve capacity found no evidence of an elevated FF and showed preserved responsiveness of GFR to dopamine in patients with early-stage ADPKD, suggesting that hyperfiltration may not be an early feature of the disease.^32^ In a study that included healthy controls, young patients with ADPKD exhibited lower RBF and higher FF, both of which were cross-sectionally associated with eGFR and TKV.^13^ Additional cross-sectional and longitudinal studies that assessed ADPKD cohorts have reported relationships between RBF and biomarkers of disease severity (kidney volume and GFR) or disease progression (change in kidney volume and GFR).^11^^,^^12^

Recently, a magnetic resonance imaging (MRI)-based longitudinal study indicated that lower RBF may be associated with faster kidney function decline and progression to kidney failure; however, a major limitation was that MIC and pathogenic variant type were not incorporated into these analyses.^10^ By combining comparisons with healthy controls and both cross-sectional and longitudinal analyses, this study extends previous work on kidney hemodynamics in ADPKD by showing that RBF is not only independently associated with disease severity, but also with subsequent disease progression, even after adjustment for MIC or pathogenic variant type. It further shows that adding information on RBF to the MIC substantially improves prognostic discrimination.

The hemodynamic changes observed in this study can be explained by cysts forming and enlarging, thereby distorting the surrounding tissue and compressing blood vessels.^14^ This reduces the amount of blood that can effectively be delivered to the kidney through the microvasculature. The resulting reduction in oxygen delivery and ischemia strongly activates renin-angiotensin-aldosterone system, and this activation is further amplified by non-ACE pathways, such as chymase.^33^, ^34^, ^35^ Increased angiotensin II causes predominantly efferent arteriolar constriction, which raises glomerular capillary pressure and allows a greater FF of the delivered plasma to be filtered.^36^ Because the degree of hyperfiltration was not associated with kidney volume or subsequent kidney function decline, it appears to represent an early adaptive hemodynamic response rather than a predictor of disease progression.

We did not include pathogenic variant type nor Predicting Renal Outcomes in Polycystic Kidney disease score in the primary models. Genetic information is not always routinely available in clinical practice. For that reason, the Predicting Renal Outcomes in Polycystic Kidney disease score is often unavailable. This issue is also relevant in older patients with ADPKD who receive their diagnosis later in life and in patients aged <35 years, for whom data on clinically relevant events, including age at onset of hypertension and urological complications, may be unreliable or unavailable because such events may not yet have occurred. Consequently, the generalizability of prediction models that rely on genetic data or the Predicting Renal Outcomes in Polycystic Kidney disease score may be limited.^37^ However, to evaluate whether inclusion of genetic data influenced the results, we performed a sensitivity analysis including pathogenic variant type.

Although RBF is modifiable, medications that influence renal hemodynamics are not prescribed specifically to modify RBF. Our study therefore focuses on its role as a prognostic marker rather than a therapeutic target. From a clinical perspective, RBF as prognostic marker may be particularly valuable in young patients or patients with borderline or intermediate-risk profiles, such as those classified as MIC 1C, in whom treatment decisions can be challenging. In both groups RBF was associated with clinical progression outcomes in univariate analyses, although none of these associations remained significant after adjustment for established clinical risk factors. The similar magnitude of the coefficients of the predictive power of RBF for the various outcomes in the younger and MIC 1C subgroups when compared with the overall population suggests that this is because of reduced power because of the lower number of subjects under investigation rather than a fundamentally different underlying mechanism in younger subjects when compared to older subjects.

In this study, RBF was derived from ERPF estimated by ^131^I-Hippuran. This approach has known limitations, for example, it is sensitive to variation in hematocrit. Nevertheless ERPF-derived RBF correlates strongly with MRI-based measurements, despite consistently lower absolute values.^38^ These differences are therefore unlikely to affect the observed associations within the study population, because the relative variation between patients is preserved. Although MRI-based techniques may provide higher absolute estimates of RBF, their applicability is limited in more advanced disease stages (eGFR ≤ 70 ml/min per 1.73 m^2^), possibly because of technical limitations related to lower blood flow in smaller renal arteries.^38^ This may also explain the high number of missing RBF measurements reported in the previous MRI-based study.^10^ Taken together, ERPF-derived RBF represents a suitable method for assessing hemodynamic associations in this cohort.

Several limitations should be considered. First, creatinine-based eGFR is assumed less accurate than iothalamate-measured GFR for estimating kidney function decline (eGFR slope), classifying rapid and slow progressors and defining the composite end point, as discussed above.^39^, ^40^, ^41^, ^42^ We decided to use eGFR slope because eGFR is the measure routinely used in clinical practice, which increases the clinical applicability of our results. Importantly, when rapid progression was assessed using mGFR instead of eGFR, essentially similar results were obtained. Second, ERPF-derived RBF measurements are susceptible to physiological confounders and are not routinely performed, which may limit their implementation alongside MIC. Third, the predominantly white European ancestry of the study population may limit generalizability to other populations.

## Conclusion

In conclusion, reduced RBF is an early feature of ADPKD and is associated with disease severity and subsequent decline in kidney function. RBF provides additional prognostic information beyond the MIC. These findings suggest that changes in kidney hemodynamics are related to disease progression in ADPKD. Similar strengths of associations were observed in younger patients and in patients with MIC 1C, although these associations were not significant because of the lower number of subjects involved. Interestingly, advanced MRI biomarkers, particularly cyst parenchyma surface area, and cyst number have also been shown to add significantly to MIC.^43^ Future studies should focus on assessing whether the observed improvements in predictive performance indeed translate into meaningful changes in clinical decision-making and patient management.

## Appendix

### List of the DIPAK Consortium

J.P.H. Drenth, R.T. Gansevoort, M.D.A. van Gastel, E. Meijer, L. Messchendorp, T. Nijenhuis, T. Bais, D.J.M. Peters, M. Salih, and S. Spijker.

## Disclosure

All the authors declare no disclosures relevant to this manuscript.

## Supplementary Material

Supplementary File **Supplementary Methods**. **Figure S1.** Schematic overview of patient selection. **Figure S2.** Pearson correlation matrix of kidney hemodynamic parameters. **Table S1.** Clinical characteristics of patients with ADPKD stratified by the rate of disease progression. **Table S2.** Subgroup analysis of patients younger than 40 years. **Table S3.** Subgroup analysis of patients with Mayo Imaging Class 1C. **Table S4.** Cross-sectional analyses with additional adjustment for pathogenic variant type. **Table S5.** Longitudinal analyses with additional adjustment for pathogenic variant type. STROBE checklist.

## Footnotes

Supplementary File (PDF)
**Supplementary Methods**. **Figure S1.** Schematic overview of patient selection. **Figure S2.** Pearson correlation matrix of kidney hemodynamic parameters. **Table S1.** Clinical characteristics of patients with ADPKD stratified by the rate of disease progression. **Table S2.** Subgroup analysis of patients younger than 40 years. **Table S3.** Subgroup analysis of patients with Mayo Imaging Class 1C. **Table S4.** Cross-sectional analyses with additional adjustment for pathogenic variant type. **Table S5.** Longitudinal analyses with additional adjustment for pathogenic variant type. STROBE checklist.

## References

[1] Lanktree M.B., Haghighi A., Guiard E. (2018). Prevalence estimates of polycystic kidney and liver disease by population sequencing. J Am Soc Nephrol.

[2] Torres V.E., Harris P.C., Pirson Y. (2007). Autosomal dominant polycystic kidney disease. Lancet.

[3] Spithoven E.M., Kramer A., Meijer E. (2014). Renal replacement therapy for autosomal dominant polycystic kidney disease (ADPKD) in Europe: prevalence and survival--an analysis of data from the ERA-EDTA Registry. Nephrol Dial Transplant.

[4] Persu A., Duyme M., Pirson Y. (2004). Comparison between siblings and twins supports a role for modifier genes in ADPKD. Kidney Int.

[5] Lanktree M.B., Guiard E., Li W. (2019). Intrafamilial variability of ADPKD. Kidney Int Rep.

[6] Torres V.E., Chapman A.B., Devuyst O. (2012). Tolvaptan in patients with autosomal dominant polycystic kidney disease. N Engl J Med.

[7] Müller R.U., Messchendorp A.L., Birn H. (2022). An update on the use of tolvaptan for autosomal dominant polycystic kidney disease: consensus statement on behalf of the ERA Working Group on Inherited Kidney Disorders, the European Rare Kidney Disease Reference Network and Polycystic Kidney Disease International. Nephrol Dial Transplant.

[8] Irazabal M.V., Rangel L.J., Bergstralh E.J. (2015). Imaging classification of autosomal dominant polycystic kidney disease: a simple model for selecting patients for clinical trials. J Am Soc Nephrol.

[9] Bais T., Geertsema P., Knol M.G.E. (2024). Validation of the mayo imaging classification system for predicting kidney outcomes in ADPKD. Clin J Am Soc Nephrol.

[10] Nowak K.L., Copeland T.P., Ku E. (2026). Renal blood flow and progression to kidney failure in ADPKD. Nephrol Dial Transplant.

[11] Torres V.E., King B.F., Chapman A.B. (2007). Magnetic resonance measurements of renal blood flow and disease progression in autosomal dominant polycystic kidney disease. Clin J Am Soc Nephrol.

[12] King B.F., Torres V.E., Brummer M.E. (2003). Magnetic resonance measurements of renal blood flow as a marker of disease severity in autosomal-dominant polycystic kidney disease11Thomas Andreoli, M.D., served as Guest Editor for this paper. Kidney Int.

[13] Meijer E., Rook M., Tent H. (2010). Early renal abnormalities in autosomal dominant polycystic kidney disease. Clin J Am Soc Nephrol.

[14] Ecder T., Schrier R.W. (2009). Cardiovascular abnormalities in autosomal-dominant polycystic kidney disease. Nat Rev Nephrol.

[15] Helal I., Reed B., McFann K. (2011). Glomerular hyperfiltration and renal progression in children with autosomal dominant polycystic kidney disease. Clin J Am Soc Nephrol CJASN.

[16] Devuyst O., Ahn C., Barten T.R.M., Brosnahan G., Cadnapaphornchai M.A., Chapman A.B. (2025). KDIGO 2025 clinical practice guideline for the evaluation, management, and treatment of autosomal dominant polycystic kidney disease (ADPKD). Kidney Int.

[17] Pei Y., Obaji J., Dupuis A. (2009). Unified criteria for ultrasonographic diagnosis of ADPKD. J Am Soc Nephrol.

[18] Posthumus A.M., Knobbe T.J., Kremer D. (2025). TransplantLines, a biobank and cohort study of solid organ transplant recipients and donors. Eur J Epidemiol.

[19] Eisenga M.F., Gomes-Neto A.W., Van Londen M. (2018). Rationale and design of TransplantLines: a prospective cohort study and biobank of solid organ transplant recipients. BMJ Open.

[20] van Gastel M.D.A., Edwards M.E., Torres V.E., Erickson B.J., Gansevoort R.T., Kline T.L. (2019). Automatic measurement of kidney and liver volumes from MR images of patients affected by autosomal dominant polycystic kidney disease. J Am Soc Nephrol.

[21] Donker A.J., van der Hem G.K., Sluiter W.J., Beekhuis H. (1977). A radioisotope method for simultaneous determination of the glomerular filtration rate and the effective renal plasma flow. Neth J Med.

[22] Levey A.S., Stevens L.A., Schmid C.H. (2009). A new equation to estimate glomerular filtration rate. Ann Intern Med.

[23] Gansevoort R.T., Anders H.J., Cozzolino M. (2023). What should European nephrology do with the new CKD-EPI equation?. Nephrol Dial Transplant.

[24] Mosteller R.D. (1987). Simplified calculation of body-surface area. N Engl J Med.

[25] Kokholm G. (1990). Simultaneous measurements of blood pH, *p* CO_2_, *p* O_2_ and concentrations of hemoglobin and its derivates-A multicenter study. Scand J Clin Lab Investig.

[26] Cersosimo E., YueJuan Q., Baskoy G. (2025). Effect of dapagliflozin on renal haemodynamics in hyperfiltering T2D patients. Diabetes Obes Metab.

[27] Cornec-Le Gall E., Alam A., Perrone R.D. (2019). Autosomal dominant polycystic kidney disease. Lancet.

[28] Pencina M.J., D’Agostino R.B., D’Agostino R.B., Vasan R.S. (2008). Evaluating the added predictive ability of a new marker: from area under the ROC curve to reclassification and beyond. Stat Med.

[29] Harrell F.E., Califf R.M., Pryor D.B., Lee K.L., Rosati R.A. (1982). Evaluating the yield of medical tests. JAMA.

[30] Franz K.A., Reubi F.C. (1983). Rate of functional deterioration in polycystic kidney disease. Kidney Int.

[31] Wong H., Vivian L., Weiler G., Filler G. (2004). Patients with autosomal dominant polycystic kidney disease hyperfiltrate early in their disease. Am J Kidney Dis.

[32] Messchendorp A.L., Van Londen M., Taylor J.M. (2018). Kidney function reserve capacity in early and later stage autosomal dominant polycystic kidney disease. Clin J Am Soc Nephrol.

[33] Saigusa T., Dang Y., Bunni M.A. (2015). Activation of the intrarenal renin-angiotensin-system in murine polycystic kidney disease. Physiol Rep.

[34] Ecder T., Schrier R.W. (2001). Hypertension in autosomal-dominant polycystic kidney disease: early occurrence and unique aspects. J Am Soc Nephrol.

[35] McPherson E.A., Luo Z., Brown R.A. (2004). Chymase-like angiotensin II–generating activity in end-stage human autosomal dominant polycystic kidney disease. J Am Soc Nephrol.

[36] Hall J.E., Guyton A.C., Jackson T.E., Coleman T.G., Lohmeier T.E., Trippodo N.C. (1977). Control of glomerular filtration rate by renin-angiotensin system. Am J Physiol Ren Physiol.

[37] Cornec-Le Gall E., Audrézet M.P., Rousseau A. (2016). The PROPKD score: a new algorithm to predict renal survival in autosomal dominant polycystic kidney disease. J Am Soc Nephrol.

[38] Spithoven E.M., Meijer E., Borns C. (2016). Feasibility of measuring renal blood flow by phase-contrast magnetic resonance imaging in patients with autosomal dominant polycystic kidney disease. Eur Radiol.

[39] Fernandez J.M., Rodriguez-Pérez J.C., Lorenzo-Medina M.M., Rodriguez-Esparragon F., Quevedo-Reina J.C., Hernandez-Socorro C.R. (2025). Longitudinal assessment of measured and estimated glomerular filtration-rate in autosomal dominant polycystic kidney disease: real practice experience. World J Nephrol.

[40] Rodríguez R.M., Luis-Lima S., Fernandez J.M. (2022). Estimated GFR in autosomal dominant polycystic kidney disease: errors of an unpredictable method. J Nephrol.

[41] Ruggenenti P., Gaspari F., Cannata A. (2012). Measuring and estimating GFR and treatment effect in ADPKD patients: results and implications of a longitudinal cohort study. PLOS One.

[42] Spithoven E.M., Meijer E., Boertien W.E. (2013). Tubular secretion of creatinine in autosomal dominant polycystic kidney disease: consequences for cross-sectional and longitudinal performance of kidney function estimating equations. Am J Kidney Dis.

[43] Gregory A.V., Chebib F.T., Poudyal B. (2023). Utility of new image-derived biomarkers for autosomal dominant polycystic kidney disease prognosis using automated instance cyst segmentation. Kidney Int.

