# Supplementary material for: Renal Blood Flow as Marker for Disease Progression in ADPKD
Source: Kidney Int Rep. 2026 Sep 1;11(12):107050. doi: 10.1016/j.ekir.2026.107050 (PMC13694765; doi:10.1016/j.ekir.2026.107050)
Supplement: Supplementary file 1 [file mmc1.pdf]

Supplementary Material

Table of contents

1. Supplementary Methods: Description of kidney function measurements.

2. Supplementary Table 1: Clinical characteristics of patients with ADPKD stratified by the rate of disease progression.

3. Supplementary Table 2: Subgroup analysis of patients younger than 40 years.

4. Supplementary Table 3: Subgroup analysis of patients with Mayo Imaging Class 1C.

5. Supplementary Table 4: Cross-sectional analyses with additional adjustment for pathogenic variant type.

6. Supplementary Table 5: Longitudinal analyses with additional adjustment for pathogenic variant type.

7. Supplementary Figure 1: Schematic overview of patient selection.

8. Supplementary Figure 2: Pearson correlation matrix of kidney hemodynamic parameters.

9. STROBE checklist.

Supplementary information is available at KI Reports' website.

Supplementary Methods. Description of kidney function measurements

Patients arrived in a fasting state and were allowed to drink ad libitum. mGFR and ERPF were determined by constant infusion of ^{125}I-iothalamate and ^{131}I-hippuran. Prior to the procedure, patients received Lugol's solution to block thyroidal uptake of radioactive iodine. An initial priming infusion of 20 mL containing both tracers was administered intravenously. This was followed by a continuous infusion of both tracers using solutions with concentrations of 0.0075 MBq/mL for ^{125}I-iothalamate and 0.02 MBq/mL for ^{131}I-hippuran. Infusion rates were adjusted according to kidney function, with rates of 12 mL/h for serum creatinine <100 µmol/L, 9 mL/h for 100–200 µmol/L, and 6 mL/h for >200 µmol/L.

Following an equilibration phase of 90 minutes, during which plasma tracer concentrations stabilized, serial blood samples were collected at hourly intervals throughout the remainder of the procedure. Urine was collected as naturally voided samples during two consecutive clearance periods of 2 hours each.

Calculations

Under steady-state conditions, the infusion-based method $\frac{I_{hipp} \cdot V_I}{P_{hipp}}$ was used for ERPF when ERPF exceeded 100 mL/min. When ERPF was below 100 mL/min, urinary clearance $\frac{(U_{hipp} \cdot V)}{P_{hipp}}$ was used.

where $U \cdot V$ represents urinary tracer excretion, $I \cdot V_I$ the tracer infusion rate, and P the plasma tracer concentration measured under steady-state conditions.

mGFR was calculated as $\frac{(U_{iot} \cdot V)}{P_{iot}}$.

To account for incomplete bladder emptying, mGFR was corrected using the ratio between infusion-based and urinary hippuran clearance.

Supplementary Table 1. Clinical characteristics of patients with ADPKD stratified by the rate of disease progression, defined by annual eGFR decline.

Variable	Overall (n=238)	Rate of disease progression: Rapid (n=112)	Rate of disease progression: Slow (n=126)
Follow up time, yr, mean ± SD	5.6 ± 3.5	4.8 ± 3.2	6.3 ± 3.6
eGFR slope, mL/min/1.73m^2 per year, mean ± SD	-2.79 ± 1.27	-3.83 ± 0.75	-1.86 ± 0.85
eGFR measurements, median (IQR)	6 (3-10)	5 (3-8)	8 (3-10)
HtTKV slope, %/yr, mean ± SD	4.89 ± 3.73	6.21 ± 3.73	3.93 ± 3.46
HtTKV measurements, median (IQR)	2 (2-3)	2 (2-3)	3 (2-3)
Kidney failure, No. (%)	51 (21.4)	46 (41.1)	5 (4.0)
Kidney failure or 30% eGFR loss, No. (%)	111 (46.6)	83 (74.1)	28 (22.2)

Rate of disease progression was defined according to the annual decline in estimated glomerular filtration rate (eGFR): rapid progression ≥3 mL/min/1.73 m² per year.
Kidney failure was defined as initiation of dialysis, kidney transplantation or an eGFR ≤ 15 mL/min/1.73 m².
Data are presented as number (percentage) or mean ± SD, unless otherwise specified.
HtTKV slope was available in 147 patients overall (62 in the rapid progression group and 85 in the slow progression group).
Abbreviations: eGFR, estimated glomerular filtration rate; HtTKV, height-adjusted total kidney volume.

Supplementary Table 2: Longitudinal associations of RBF and FF with kidney function outcomes among patients with ADPKD younger than 40 years (n=84).

Variable	eGFR Slope		Rapidly Progressive Disease		Kidney Failure or 30% eGFR Decline	
	St. β-estimate (95% CI)	*P* value	OR (95% CI)	*P* value	HR (95% CI)	*P* value
Primary cohort (n=84)						
Model 1						
RBF	0.20 (-0.04 to 0.53)	0.09	0.48 (0.26 to 0.91)	0.02	0.48 (0.26 to 0.89)	0.02
FF	-0.09 (-0.32 to 0.14)	0.5	1.17 (0.75 to 1.85)	0.5	1.03 (0.72 to 1.47)	0.9
Model 2						
RBF	0.26 (-0.07 to 0.72)	0.1	0.42 (0.16 to 1.09)	0.07	0.64 (0.27 to 1.51)	0.3
FF	-0.08 (-0.32 to 0.16)	0.5	1.17 (0.67 to 2.01)	0.6	0.98 (0.61 to 1.60)	0.9
Model 3						
RBF	0.16 (-0.19 to 0.59)	0.3	0.52 (0.20 to 1.36)	0.2	0.63 (0.27 to 1.45)	0.3
FF	-0.13 (-0.36 to 0.11)	0.3	1.33 (0.75 to 2.37)	0.3	1.06 (0.63 to 1.77)	0.8

Model 1, univariate. Model 2, adjusted for sex, baseline age, eGFR at baseline and Mayo Imaging Classification. Model 3, identical to Model 2 with substitution of baseline mGFR for baseline eGFR. All continuous variables were z-standardised. Standardized β represents the SD change in the outcome per 1-SD higher RBF or FF; a positive β for eGFR slope indicates slower decline. ORs and HRs are reported per 1-SD higher RBF or FF; values <1 indicate lower odds or risk.

Abbreviations: eGFR, estimated glomerular filtration rate; RBF, Renal Blood Flow; FF, Filtration Fraction; mGFR, measured glomerular filtration rate; CI, confidence interval; OR, odds ratio; HR, hazard ratio.

Supplementary Table 3: Longitudinal associations of RBF and FF with kidney function outcomes among patients with ADPKD with Mayo Imaging Class 1C (n=77).

Variable	eGFR Slope		Rapidly Progressive Disease		Kidney Failure or 30% eGFR Decline	
	St. β-estimate (95% CI)	*P* value	OR (95% CI)	*P* value	HR (95% CI)	*P* value
Primary cohort (n=77)						
Model 1						
RBF	0.46 (0.23 to 0.60)	<0.001	0.47 (0.28 to 0.81)	0.01	0.20 (0.12 to 0.35)	<0.001
FF	0.11 (-0.09 to 0.31)	0.3	0.67 (0.41 to 1.08)	0.1	0.73 (0.54 to 1.00)	0.051
Model 2						
RBF	0.35 (-0.12 to 0.75)	0.2	0.76 (0.23 to 2.48)	0.6	0.44 (0.18 to 1.08)	0.1
FF	-0.00 (-0.19 to 0.19)	1.0	0.77 (0.45 to 1.30)	0.3	0.83 (0.58 to 1.18)	0.3
Model 3						
RBF	0.06 (-0.45 to 0.55)	0.8	1.55 (0.38 to 6.42)	0.5	0.68 (0.22 to 2.17)	0.7
FF	-0.07 (-0.25 to 0.13)	0.5	0.87 (0.51 to 1.49)	0.6	1.00 (0.71 to 1.42)	1.0

Model 1, univariate. Model 2, adjusted for sex, baseline age, eGFR at baseline and Mayo Imaging Classification. Model 3, identical to Model 2 with substitution of baseline mGFR for baseline eGFR. All continuous variables were z-standardised. Standardized β represents the SD change in the outcome per 1-SD higher RBF or FF; a positive β for eGFR slope indicates slower decline. ORs and HRs are reported per 1-SD higher RBF or FF; values <1 indicate lower odds or risk.

Abbreviations: eGFR, estimated glomerular filtration rate; RBF, Renal Blood Flow; FF, Filtration Fraction; mGFR, measured glomerular filtration rate; CI, confidence interval; OR, odds ratio; HR, hazard ratio.

Supplementary Table 4. Cross-sectional associations of RBF and FF with kidney function measures in patients with ADPKD, additionally adjusted for pathogenic variant type.

Variable	Model	eGFR		mGFR		HtTKV	
		St. β-estimate (95% CI)	*P* value	St. β-estimate (95% CI)	*P* value	St. β-estimate (95% CI)	*P* value
RBF	1	0.85 (0.76 to 0.90)	<0.001	0.86 (0.78 to 0.91)	<0.001	-0.36 (-0.44 to -0.20)	<0.001
	2	0.69 (0.60 to 0.75)	<0.001	0.74 (0.65 to 0.80)	<0.001	-0.22 (-0.31 to -0.08)	0.001
FF	1	0.27 (0.15 to 0.40)	<0.001	0.36 (0.24 to 0.48)	<0.001	-0.13 (-0.24 to 0.01)	0.07
	2	0.11 (0.03 to 0.20)	0.01	0.21 (0.13 to 0.30)	<0.001	-0.09 (-0.16 to 0.01)	0.07

Model 1, univariate. Model 2, adjusted for sex, baseline age, Mayo imaging classification and pathogenic variant type. All continuous variables were z-standardised. Standardized β represents the SD change in the outcome per 1-SD higher RBF or FF.

Abbreviations: eGFR, estimated glomerular filtration rate; HtTKV, height-adjusted total kidney volume; mGFR, measured glomerular filtration rate; RBF, renal blood flow; FF, filtration fraction; CI, confidence interval.

Supplementary Table 5. Longitudinal associations of RBF and FF with kidney function outcomes in patients with ADPKD, additionally adjusted for pathogenic variant type.

Variable	eGFR Slope		Rapidly Progressive Disease		Kidney Failure or 30% eGFR Decline	
	St. β-estimate (95% CI)	*P* value	OR (95% CI)	*P* value	HR (95% CI)	*P* value
Primary cohort (n=238)						
Model 1						
RBF	0.29 (0.04 to 0.54)	0.02	0.36 (0.18 to 0.76)	0.01	0.37 (0.22 to 0.65)	<0.001
FF	-0.02 (-0.14 to 0.10)	0.8	1.12 (0.79 to 1.59)	0.5	0.96 (0.76 to 1.21)	0.7
Model 2						
RBF	0.10 (-0.16 to 0.36)	0.5	0.47 (0.22 to 1.00)	0.049	0.50 (0.28 to 0.88)	0.02
FF	-0.07 (-0.19 to 0.06)	0.3	1.36 (0.93 to 2.00)	0.1	1.12 (0.91 to 1.45)	0.3

Model 1, adjusted for sex, baseline age, eGFR at baseline, Mayo Imaging Classification and pathogenic variant type. Model 2, identical to Model 1 with substitution of baseline mGFR for baseline eGFR. All continuous variables were z-standardised. Standardized β represents the SD change in the outcome per 1-SD higher RBF or FF; a positive β for eGFR slope indicates slower decline. ORs and HRs are reported per 1-SD higher RBF or FF; values <1 indicate lower odds or risk.

Abbreviations: eGFR, estimated glomerular filtration rate; RBF, Renal Blood Flow; FF, Filtration Fraction; mGFR, measured glomerular filtration rate; CI, confidence interval; OR, odds ratio; HR, hazard ratio.

Supplementary Figure 1. Schematic overview of patient selection.

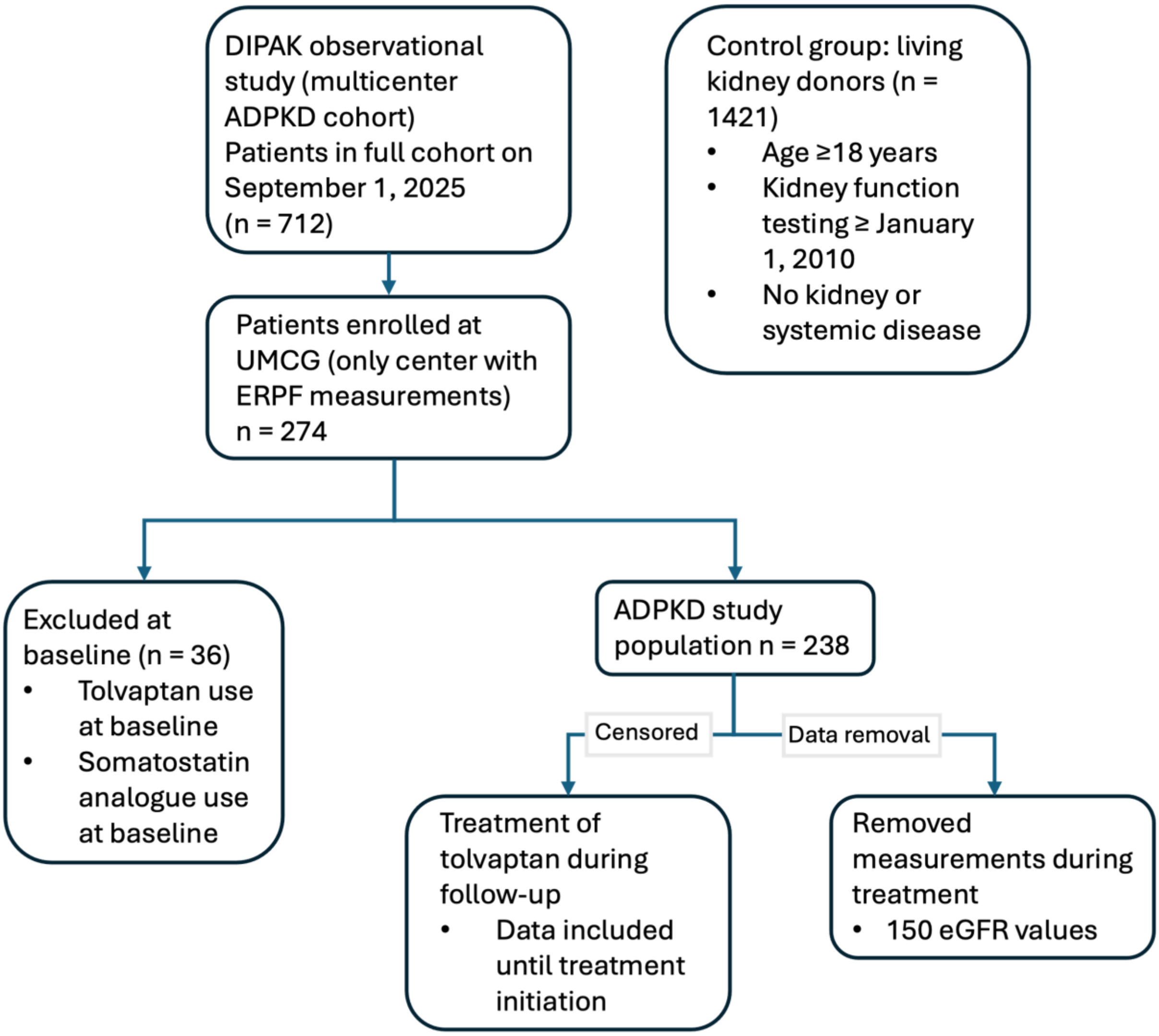

Supplementary Figure 2. Pearson correlation matrix of kidney hemodynamic parameters.

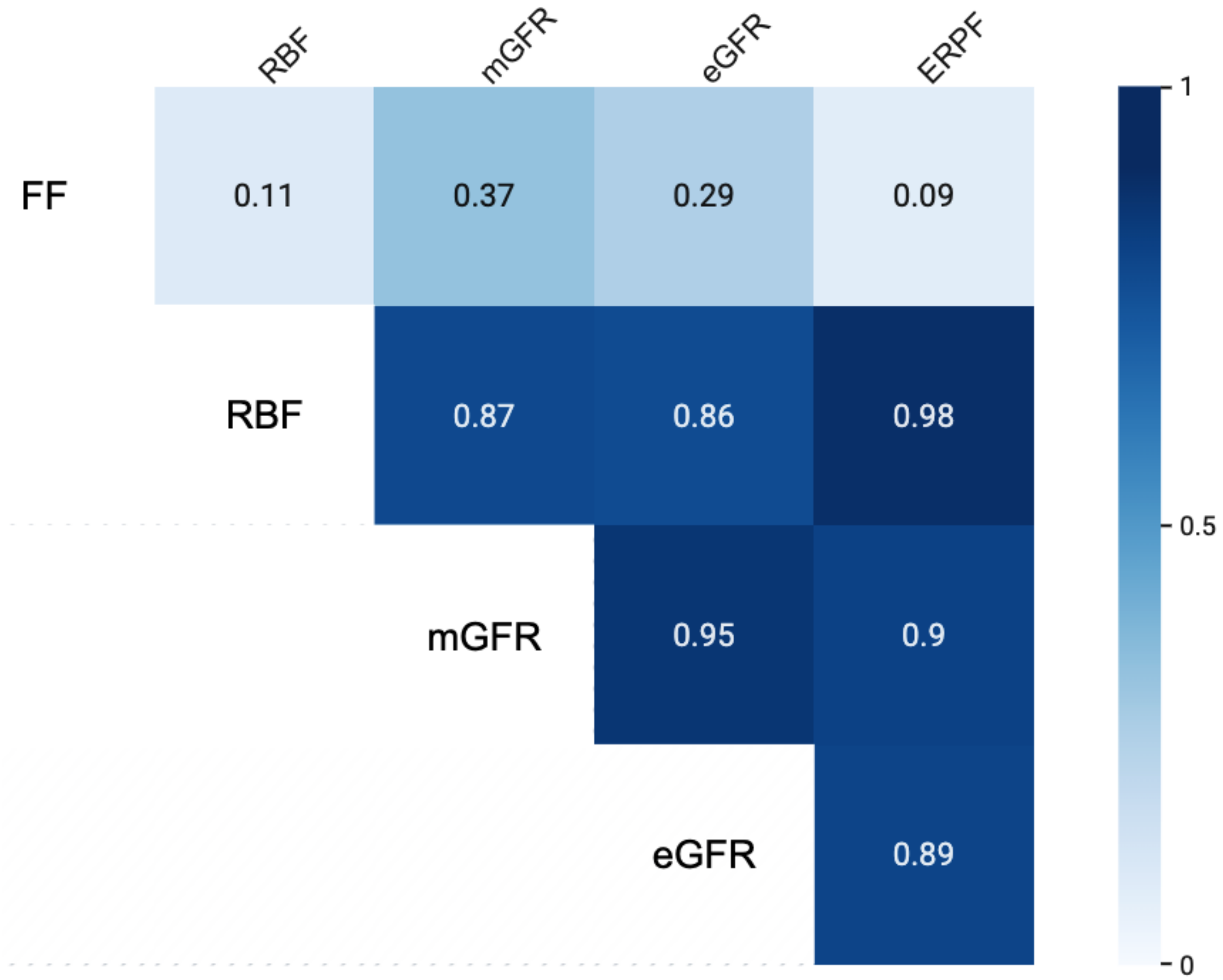

STROBE Statement—Checklist of items that should be included in reports of ***cohort studies***

	Item No	Recommendation	Page No
**Title and abstract**	1	(*a*) Indicate the study's design with a commonly used term in the title or the abstract	1/2
		(*b*) Provide in the abstract an informative and balanced summary of what was done and what was found	2
**Introduction**			
Background/rationale	2	Explain the scientific background and rationale for the investigation being reported	3/4
Objectives	3	State specific objectives, including any prespecified hypotheses	4
**Methods**			
Study design	4	Present key elements of study design early in the paper	6-8
Setting	5	Describe the setting, locations, and relevant dates, including periods of recruitment, exposure, follow-up, and data collection	5
Participants	6	(*a*) Give the eligibility criteria, and the sources and methods of selection of participants. Describe methods of follow-up	5
		(*b*) For matched studies, give matching criteria and number of exposed and unexposed	-
Variables	7	Clearly define all outcomes, exposures, predictors, potential confounders, and effect modifiers. Give diagnostic criteria, if applicable	8
Data sources/ measurement	8*	For each variable of interest, give sources of data and details of methods of assessment (measurement). Describe comparability of assessment methods if there is more than one group	6
Bias	9	Describe any efforts to address potential sources of bias	5
Study size	10	Explain how the study size was arrived at	5
Quantitative variables	11	Explain how quantitative variables were handled in the analyses. If applicable, describe which groupings were chosen and why	7
Statistical methods	12	(*a*) Describe all statistical methods, including those used to control for confounding	7-8
		(*b*) Describe any methods used to examine subgroups and interactions	9
		(*c*) Explain how missing data were addressed	7
		(*d*) If applicable, explain how loss to follow-up was addressed	-
		(*e*) Describe any sensitivity analyses	9/14
**Results**			
Participants	13*	(a) Report numbers of individuals at each stage of study—eg numbers potentially eligible, examined for eligibility, confirmed eligible, included in the study, completing follow-up, and analysed	10
		(b) Give reasons for non-participation at each stage	Sup
		(c) Consider use of a flow diagram	Sup
Descriptive data	14*	(a) Give characteristics of study participants (eg demographic, clinical, social) and information on exposures and potential confounders	10
		(b) Indicate number of participants with missing data for each variable of interest	10
		(c) Summarise follow-up time (eg, average and total amount)	11
Outcome data	15*	Report numbers of outcome events or summary measures over time	11-14

Main results	16	(*a*) Give unadjusted estimates and, if applicable, confounder-adjusted estimates and their precision (eg, 95% confidence interval). Make clear which confounders were adjusted for and why they were included	11-14
		(*b*) Report category boundaries when continuous variables were categorized	11-14
		(*c*) If relevant, consider translating estimates of relative risk into absolute risk for a meaningful time period	-
Other analyses	17	Report other analyses done—eg analyses of subgroups and interactions, and sensitivity analyses	13/14
**Discussion**			
Key results	18	Summarise key results with reference to study objectives	15
Limitations	19	Discuss limitations of the study, taking into account sources of potential bias or imprecision. Discuss both direction and magnitude of any potential bias	18
Interpretation	20	Give a cautious overall interpretation of results considering objectives, limitations, multiplicity of analyses, results from similar studies, and other relevant evidence	15-18
Generalisability	21	Discuss the generalisability (external validity) of the study results	18
**Other information**			
Funding	22	Give the source of funding and the role of the funders for the present study and, if applicable, for the original study on which the present article is based	19

*Give information separately for exposed and unexposed groups.

Note: An Explanation and Elaboration article discusses each checklist item and gives methodological background and published examples of transparent reporting. The STROBE checklist is best used in conjunction with this article (freely available on the Web sites of PLoS Medicine at http://www.plosmedicine.org/, Annals of Internal Medicine at http://www.annals.org/, and Epidemiology at http://www.epidem.com/). Information on the STROBE Initiative is available at http://www.strobe-statement.org.
